# Relationship of leisure-time and household physical activity level and type with cardiovascular disease: secondary analysis of the Takashima Study data

**DOI:** 10.1186/s12872-022-02569-x

**Published:** 2022-03-30

**Authors:** Hiroaki Iwase, Sachiko Tanaka-Mizuno, Naoyuki Takashima, Aya Kadota, Kenji Matsui, Yasuyuki Nakamaura, Katsuyuki Miura, Hirotsugu Ueshima, Yoshikuni Kita

**Affiliations:** 1 Department of Public Health, Shiga University of Medical Science, Otsu, Japan; 2 Department of Physical Therapy, Faculty of Rehabilitation, Kobe International University, 9-1-6 Koyocho-naka, Higashinada-ku, Kobe, Hyogo 658-0032 Japan; 3 Department of Digital Health and Epidemiology, Graduate School of Medicine and Public Health, Kyoto University, Kyoto, Japan; 4 Department of Public Health, Kindai University Faculty of Medicine, Osaka-Sayama, Japan; 5 Center for Epidemiologic Research in Asia, Shiga University of Medical Science, Otsu, Japan; 6 Center for Research Administration and Support, National Cancer Center, Tokyo, Japan; 7 Yamashina Racto Clinic and Medical Examination Center, Kyoto, Japan; 8 Faculty of Nursing Science, Tsuruga Nursing University, Tsuruga, Japan

**Keywords:** Leisure-time physical activity, Household physical activity, Cardiovascular disease, Cohort study, General population, Japanese

## Abstract

**Background:**

High levels of participation in leisure-time and household physical activity lower the risk of cardiovascular disease (CVD), although it is unclear whether the number of activity types is related to new-onset CVD. We aimed to evaluate the effect of the amount of leisure-time physical activity and the number of types of leisure-time physical activities on the risk of CVD incidence.

**Methods:**

From 2002 to 2003, 3,741 participants without any history of CVD participated in the Takashima Study. Data on the amount of leisure-time and household physical activity and the types of leisure-time and household physical activity were obtained from a self-administered questionnaire. Hazard ratios for CVD (acute myocardial infarction and stroke) incidence (follow-up data from 2002 to 2013), according to the participation level and number of activity types, were calculated using Cox proportional hazards models.

**Results:**

The mean age of the subjects was 58.7 ± 13.1 years. During the mean follow-up period of 8.0 ± 1.1 years, 92 participants developed CVD. An inverse dose–response relationship was noted between the amount of leisure-time and household physical activity and CVD events. After adjusting for baseline characteristics, lifestyle-related diseases, and the amount of physical activity other than leisure-time and household, the risk of CVD onset was compared by dividing the participants into two groups by the level of participation; the highest activity group had an adjusted hazard ratio (95% confidence interval) of 0.40 (0.20–0.82) compared to the lowest activity group. Compared to participants who engaged in 0–1 type of activity, participants who engaged in two or more types of activities had a multivariable-adjusted hazard ratio (95% confidence interval) of 0.31 (0.12–0.79).

**Conclusion:**

Increasing the amount of leisure-time and household physical activity and promoting engagement in two or more types of such activities may reduce the rate of CVD incidence in the Japanese general population.

## Background

In recent years, a low of physical activity has been recognized as a major risk factor for death [1, 2]. Since 2000, the level of participation in physical activity among Japanese people has been decreasing [3], thereby posing a significant public health burden. Therefore, there is an urgent need for the formulation of strategies that are aimed at encouraging people to participate in adequate levels of physical activity. Previously, the US Department of Health and Human Services (HHS) reported that low levels of physical activity were strongly associated with the risk of cardiovascular disease (CVD) [4], and that there exists a dose–response relationship between the physical activity level and the CVD risk [4–7]. The HHS guidelines recommended that adults should do at least 150 min (2 h and 30 min) to 300 min (5 h) a week of moderate-intensity, or 75 min (1 h and 15 min) to 150 min (2 h and 30 min) a week of vigorous-intensity aerobic physical activity, or an equivalent combination of moderate- and vigorous-intensity aerobic activity [8].

In general, physical activity is a concept consisting of occupational physical activity (work-related), transportation (walking or biking for the purposes of going somewhere), household physical activity, and leisure-time physical activity.

Currently, the amount of physical activity associated with work has decreased due to the mechanization of the working environment, and recent physical activity epidemiological studies have focused more attention on indicators related to leisure activities such as exercise, sports, hobbies, and volunteering, rather than physical activity by work. It has been reported that the risk of CVD onset is reduced even with relatively light intense physical activity such as walking and jogging [9, 10].

In addition, since there are many housework activities that occupy the activity time of the day, attention is also focused on housework activities [11–13]. The more housework activities, the lower the risk of total death, CVD death, and cancer death. Has also been shown to contribute to health benefits [14, 15].

Most previous studies on leisure-time and household activities in this context focused predominantly on the evaluation of the amount of physical activity. One study showed that individuals who engage in four or more types of leisure-time activities have a lower risk of dementia than those who do not engage in such activities or participate in only one activity type [16]. However, no study has focused on the effect of the number of types of leisure-time and household physical activities that a person engages in in relation to the effect on the CVD risk.

This study was conducted to longitudinally evaluate the association between the amount of leisure-time and household physical activity and the number of types of leisure-time and household physical activity and the risk of developing CVD in the general Japanese population.

## Methods

### Study design and population

The Takashima Study is a cohort study that investigated the relationships among the lifestyle, genetic factors, and lifestyle-related diseases of the general population residing in Takashima city, Shiga Prefecture, Japan [17, 18]. The participants of the present study were recruited from the aforementioned study after obtaining written informed consent for participation, and had all undergone an annual health checkup. A total of 3,741 participants (1,343 men, 2,398 women), selected from the original group of 4,653 participants of the Takashima Study who completed the baseline survey between 2002 and 2003, were enrolled for the analysis in this study. Participants with a history of cerebrovascular disease, heart disease, and cancer (471 people), those lacking data on physical activity (260 people), and those with less than 1 h of sleep, and those with more than 24 h of physical activity per day (181 people) were excluded. This study was conducted in adherence with the tenets of the Declaration of Helsinki and approved by the Institutional Review Boards of Shiga University of Medical Science and Tsuruga Nursing University (Shiga University of Medical Science approval number: G2005-103, Tsuruga Nursing University approval number: 19002).

### Outcomes

The development of CVD was the primary outcome and the occurrence of acute myocardial infarction (AMI) and stroke was the secondary outcome. Note that stroke and AMI were recorded separately. In this study, CVD was defined as AMI plus stroke. The vital status of the participants was determined from the basic resident register of the local government. First-ever CVD events were identified through the same case-finding system established the Takashima Cardio-cerebrovascular Disease Registry System, which includes data on all Takashima residents. Details of the case-finding methods, registration process, and diagnostic criteria are presented elsewhere [18, 19]. In brief, we used multiple case-finding sources for case ascertainment, including hospital records and emergency ambulance service records. The AMI and stroke diagnostic criteria followed those employed by the Monitoring System for Cardiovascular Disease, as commissioned by the Ministry of Health and Welfare, Japan; these criteria are in accordance with the World Health Organization’s Monitoring of Trends and Determinants in Cardiovascular Disease project [19, 20]. Registered AMI events were validated based on the patients’ medical history, clinical symptoms, electrocardiography findings, as well as cardiac enzyme levels. In patients with out-of-hospital cardiac death, data on electrocardiography findings and cardiac enzyme levels were often not available. In such cases, we base-registered the patients’ location and symptoms at onset, and their history of coronary heart disease. Stroke was defined as the sudden onset of neurological symptoms that continued for a minimum of 24 h or resulted in death. The case definitions of stroke corresponded to the International Classification of Diseases (ICD)-10 codes I60.0–61.9 and I63.0–63.9, whereas those of AMI corresponded to the codes ICD10 I21.0–21.9. CVD events were defined as a combination of stroke and AMI events.

### Physical activity measurement

The primary exposure factor used for the purpose of assessment in this study was leisure-time and household physical activity. The data related to leisure-time and household physical activity that were utilized in this study were obtained from the Japan Arteriosclerosis Longitudinal Study Physical Activity Questionnaire (JALSPAQ) [21, 22] that was used in the Japan Arteriosclerosis Longitudinal Study [23]. The questionnaire consists of items related to sleep, occupation (work-related), transportation (walking or bicycling to work or shopping), household, and leisure time physical activity. For leisure-time physical activities, we asked for up to eight free-descriptive (ie, "open-ended") answers. Regarding exercise, we asked you to list regular exercise at least once a month, for a total of 60 min or more per month. For each leisure activity, we asked about the number of days they participated in each activity during the month and the amount of time they spent exercising per day. Each type of activity was assigned a metabolic equivalent (MET) value and plotted on a MET table of physical activity [24]. Other items included activities that did not require much physical activity, such as watching TV, listening to music, and operating a computer. The values obtained were then multiplied by the duration of engagement in each activity for the calculation of MET-hours/day based on the sums obtained. Leisure-time physical activities were defined as a combination of exercise, non-exercise leisure activities. Although the questionnaire was self-administered, those who required assistance in filling it out received assistance from their nurses, who provided an oral explanation of the questionnaire or read the questionnaire out loud.

### Biochemical and physical examinations

Blood pressure at baseline was measured twice by trained observers using a standard electrical sphygmomanometer BP103iII (Omron Health Care, Kyoto) applied to the right arm of seated participants after at least 5 min at rest. Body mass index (BMI) was measured with a height scale and a weight scale and calculated as the weight obtained divided by the square of the height (kg/m^2^). A self-administered questionnaire, including questions on lifestyle, clinical history, family history, smoking and alcohol drinking habit, was used and verified by trained observers.

At the baseline survey, non-fasting blood samples were collected. Glycated hemoglobin (HbA1c [JDS]) was measured by latex agglutination immunoassay. The HbA1c (NGSP) was calculated as 1.02 × HbA1c (JDS) + 0.25 [25]. Moreover, serum triglycerides, total cholesterol, and high-density lipoprotein (HDL) cholesterol were measured enzymatically.

In the analysis, hypertension was defined as the use of antihypertensive medication or BP ≥ 140/90 mmHg at baseline survey. Diabetes was defined as HbA1c (NGSP) ≥ 6.5% or use of antidiabetic medication. Dyslipidemia was defined as total cholesterol ≥ 5.69 mmol/L, taking medication for dyslipidemia, and/or a history of dyslipidemia. Smoking and drinking habits were categorized as none, past, and current.

### Statistical analysis

First, we described the baseline characteristics of the study participants. Next, we stratified the study participants into two groups based on the presence or absence of CVD. The groups were then compared using the Student’s *t*- or chi-square test. We calculated the hazard ratios and their 95% confidence intervals (CIs) for CVD onset using Cox proportional hazards models, with leisure-time and household physical activities and confounding factors. Data on the amount of leisure-time and household physical activity per day was divided into three groups: men and women, and men and women combined. In addition, the amount of leisure-time and household physical activity was divided into tertile (lowest, middle, and highest) for men and women, and men and women combined. The lowest tertile was defined as the reference group. The types of leisure-time and household physical activities were divided into two groups (0–1 type and ≥ 2 types). The group with 0–1 type of leisure-time and household physical activity was defined as the reference group. Furthermore, the types of leisure-time and household activities (0–1 type and ≥ 2 types) and the tertile of leisure-time and household physical activities (lowest, middle, and highest) were combined and evaluated. Leisure time and household activity was then stratified into two groups, and hazard ratios and 95% confidence intervals for type of leisure-time and household physical activity and CVD incidence were calculated by Cox proportional hazards model, adjusting for confounding factors. In the Cox proportional hazards models, the following factors were considered in multivariable models as confounding factors: sex and age adjusted model + adjusted for smoking (currently, past, never), drinking (currently, past, never), job (y/n), BMI, hypertension (y/n), dyslipidemia(y/n), diabetes (y/n), and physical activity other than during leisure time (sleep, occupation activity, transportation, other). Additionally, sex- and age- adjusted models were also conducted as for reference. All of these variables, with the exception of the follow-up period, used information that was assessed at baseline. All the analyses were first performed for the total population, following which sex-stratified analyses were conducted. Both the primary outcome (CVD development) and secondary outcome (stroke and AMI development) were evaluated. Sex-stratified analyses were performed separately. As the number of AMI events was small in this study, we could not analyze the relationship between leisure-time and household physical activities and AMI onset. All analyses were performed using SPSS version 22 (IBM Japan, Tokyo). The level of significance was set at 5% on both sides.

## Results

### Patient characteristics

The mean age of the subjects at the time of the baseline survey was 58.7 ± 13.1 years (62.1 ± 12.5 years for men and 56.8 ± 13.0 years for women). During the follow-up period (mean follow-up, 8.0 ± 1.1 years), 92 subjects developed CVD (73 with stroke and 19 with AMI).

Table 1 shows the characteristics of the participants at the baseline, by sex. The men participants had a higher mean age, BMI, systolic and diastolic blood pressure (BP), triglyceride (TG) level, and HbA1c level, and showed a stronger tendency to smoke, consume alcohol, have hypertension and diabetes, and to work. The women participants had higher levels of high-density lipoprotein cholesterol (HDL-C) and total cholesterol than the men participants, and showed a stronger tendency to have hyperlipidemia.

**Table 1:** Characteristics of participants by gender at baseline (2002–2003), Takashima Study, Japan

	Total		Men		Women		*P* value
	n = 3,741		n = 1,343		n = 2,398		
Age (years)	58.7	(13.1)	62.1	(12.5)	56.8	(13.0)	< 0.001
BMI (kg/m^2^)	23.0	(3.0)	23.4	(2.9)	22.8	(3.0)	< 0.001
Systolic BP (mmHg)	131.5	(21.3)	136.2	(20.7)	128.8	(21.1)	< 0.001
Diastolic BP (mmHg)	78.2	(11.9)	82.1	(11.5)	76.1	(11.6)	< 0.001
HDL-cholesterol (mmol/L)	1.5	(0.4)	1.4	(0.4)	1.6	(0.4)	< 0.001
Total cholesterol (mmol/L)	5.4	(0.9)	5.2	(0.9)	5.5	(0.9)	< 0.001
Triglyceride (mmol/L)	1.1	(0.6)	1.3	(0.8)	1.0	(0.5)	< 0.001
HbA1c (%)	5.0	(0.6)	5.1	(0.7)	5.0	(0.6)	< 0.001
Smoking rate (%)	16.5		38.2		4.4		< 0.001
Alcohol consumption rate (%)	31.4		53.4		19.1		< 0.001
Hypertension treatment (%)*	18.9		22.3		17.0		< 0.001
Diabetes treatment (%) *	4.2		6.5		2.9		< 0.001
Dyslipidemia treatment (%) *	11.5		7.6		13.7		< 0.001
Employment rate (%)	49.1		57.2		44.5		< 0.001
Follow-up period (years)	8.0	(1.1)	7.9	(1.3)	8.1	(1.0)	< 0.001
Amount of physical activity (METs-hr/day)	36.5	(5.7)	35.5	(6.2)	37.0	(5.3)	< 0.001
Sleep	7.3	(1.2)	7.6	(1.3)	7.2	(1.1)	< 0.001
Occupation activity	6.4	(8.0)	8.9	(9.3)	5.0	(6.8)	< 0.001
Transportation*	2.5	(2.8)	2.6	(3.0)	2.4	(2.7)	0.091
Household	6.3	(6.2)	1.1	(2.7)	9.2	(5.8)	< 0.001
Leisure	2.3	(4.5)	2.6	(5.2)	2.1	(4.1)	0.001
Other	11.6	(4.4)	12.6	(4.5)	11.1	(4.2)	< 0.001
No. of types of leisure-time and household activities, no. of people (%)							< 0.001
0 types	377	(10.1)	338	(25.2)	39	(1.6)	
1 type	1,321	(35.3)	443	(33.0)	888	(37.0)	
2 type	1,161	(31.0)	314	(23.4)	847	(35.3)	
3 or more types	882	(23.6)	258	(18.4)	624	(26.1)	

Data are shown as mean (standard deviation) and %

*MET* metabolic equivalent; *BMI* body mass index; *BP* blood pressure; *HDL* high-density lipoprotein; *HbA1c* glcyated hemoglobin

^*^For definitions of hypertension, diabetes, and dyslipidemia mellitus, see Methods section

^*^Transportation is assumed to be walking or bicycling to work or shopping

^*^Others include activities that do not require much physical movement, such as watching TV, listening to music, and operating a computer

Investigation of the total amount of physical activity showed that the women had higher levels of participation than the men. The breakdown of total physical activity is as follows. A larger proportion of men than women engaged in sleep, occupation (work-related), leisure and “other” physical activities, whereas the women tended to participate in greater levels of household physical activity than the men. When the number of leisure-time and household items was examined, the percentage of those who did not participate in either leisure or household was higher among men than among women. On the other hand, for one, two, and three or more types of items, the percentage of women was higher than that of men.

### Breakdown of leisure-time and household physical activities

Table 2 shows the specific activities of leisure and household. As a result, housework was the most common activity for both men and women (participation rate: 47.0% for men and 98.1% for women), followed by vegetable gardening, horticulture, and gardening (participation rate: 30.7% for men and 39.7% for women), walking (participation rate: 13.5% for men and 15.2% for women), and sports (participation rate: 14.9% for men and 13.1% for women). (Participation rate: 14.9% for men and 13.1% for women).

**Table 2:** Breakdown of leisure-time and household physical activities

	Total		Men		Women		Note
	n = 3,741		n = 1,343		n = 2,398		
Household activities	2,983	(79.7)	631	(47.0)	2,352	(98.1)	
Vegetable garden, horticulture, gardening	1,364	(36.5)	412	(30.7)	952	(39.7)	
Walking	546	(14.6)	181	(13.5)	365	(15.2)	
Sports	513	(13.7)	200	(14.9)	313	(13.1)	Grand golf, gate ball, golf, jogging, swimming, volleyball, hiking, etc
Light gymnastics	321	(8.6)	82	(6.1)	239	(10.0)	Stretching, radio calisthenics, tai chi, gymnastics, etc
Volunteer	128	(3.4)	34	(2.5)	94	(3.9)	
Literary activities	70	(1.9)	14	(1.0)	56	(2.3)	Chorus, karaoke, handicraft, painting, calligraphy, etc
Dance	37	(1.0)	7	(0.5)	30	(1.3)	Folk dance, ballroom dance, traditional japanese dance, etc
Farm work (field work)	34	(0.9)	14	(1.0)	20	(0.8)	
Others	423	(11.3)	246	(18.3)	177	(7.4)	Car washing, weekend gardening, playing with my children (grandchildren), fishing, etc

Number of people (%)

### Relationship between leisure-time and household physical activity and CVD onset

Table 3 shows the relationship between leisure-time and household physical activity and CVD onset, as well as the results of the sex-stratified Cox propo

**Table 3:** Relationship between amount of leisure-time and household physical activity and onset of circulatory disease

	Men (n = 1343)									Women (n = 2,398)								
	≤ 0.43 (METs-hr/day)		0.44–3.49 (METs-hr/day)			≥ 3.50 (METs-hr/day)				≤ 7.80 (METs-hr/day)		7.81–12.70 (METs-hr/day)			≥ 12.71 (METs-hr/day)			
	n = 449		n = 447			n = 447			Trend test	n = 800		n = 805			n = 793			Trend test
			HR	95% CI	*P-value*	HR	95% CI	*P value*	*P* value			HR	95% CI	*P* value	HR	95% CI	*P* value	*P* value
*CVD*	21			14			19			16			14			8		
Sex and age adjusted model	1.00	(Ref)	0.76	(0.39–1.50)	0.433	0.93	(0.50–1.73)	0.811	0.796	1.00	(Ref)	1.04	(0.50–2.12)	0.926	0.48	(0.21–1.12)	0.089	0.102
Multivariable adjusted model	1.00	(Ref)	0.85	(0.41–1.79)	0.670	0.64	(0.28–1.43)	0.273	0.275	1.00	(Ref)	0.83	(0.34–2.00)	0.677	0.09	(0.02–0.50)	0.006	0.010
*Stroke*	17			10			14			13			12			7		
Sex and age adjusted model	1.00	(Ref)	0.67	(0.30–1.46)	0.313	0.87	(0.43–1.77)	0.699	0.671	1.00	(Ref)	1.10	(0.50–2.42)	0.812	0.52	(0.21–1.30)	0.159	0.178
Multivariable adjusted model	1.00	(Ref)	0.82	(0.35–1.92)	0.644	0.50	(0.19–1.31)	0.156	0.159	1.00	(Ref)	0.96	(0.38–2.46)	0.937	0.08	(0.01–0.54)	0.010	0.019

	Total(n = 3,741)								
	Sum of sex lowest tertiles		Sum of sex middle tertiles			Sum of sex highest tertiles			
	n = 1249		n = 1252			n = 1240			Trend test
			HR	95% CI	*P-value*	HR	95% CI	*P* value	*P* value
*CVD*	37			28			27		
Sex and age adjusted model	1.00	(Ref)	0.88	(0.54–1.44)	0.605	0.72	(0.44–1.19)	0.201	0.202
Multivariable adjusted model	1.00	(Ref)	0.91	(0.52–1.58)	0.730	0.40	(0.20–0.82)	0.012	0.015
*Stroke*	30			22			21		
Sex and age adjusted model	1.00	(Ref)	0.85	(0.49–1.48)	0.568	0.70	(0.40–1.23)	0.215	0.214
Multivariable adjusted model	1.00	(Ref)	0.89	(0.48–1.65)	0.721	0.29	(0.13–0.68)	0.005	0.006

Sex and age adjusted model: Adjusted for sex and age

Multivariable adjusted model: sex and age adjusted model + adjusted for smoking (currently, past, never), drinking (currently, past, never, job (y/n), BMI, hypertension (y/n), dyslipidemia(y/n), diabetes (y/n), and physical activity other than during leisure time (sleep, job, transportation, other)

*CVD* cardiovascular disease; *BMI* body mass index; *MET* metabolic equivalent; *HR* hazard ratio; *CI* confidence interval

rtional hazards analyses.

In the sex-stratified examination of the data, the multivariable-adjusted hazard ratio among the women with the highest level of participation in leisure-time and household physical activity was 0.09 (95% CI: 0.02–0.50) compared to that observed in the group with the lowest level. The multivariable-adjusted hazard ratio for stroke in the women was 0.08 (95% CI: 0.01–0.54). In contrast, no dose–response relationship was observed between the level of leisure-time and household physical activity and CVD in the men. Overall, the multivariable-adjusted hazard ratio for CVD onset in the group with the highest level of engagement in leisure-time and household physical activity was 0.40 (95% CI: 0.20–0.82) compared to that in the group with the lowest level of participation. The multivariable-adjusted hazard ratio for stroke was 0.29 (95% CI: 0.13–0.68). The relationship between leisure-time and household physical activities and AMI incidence could not be analyzed due to the small number of AMI incidents.

### Relationship between the number of leisure-time and household physical activity types and CVD onset

Table 4 shows the results of the Cox proportional hazards models performed for the evaluation of the relationship between the number of leisure-time and household physical activity types and CVD onset. The sex-stratified examination of the data showed that, among the women, the group that engaged in ≥ 3 types of leisure-time and household physical activities had a hazard ratio of 0.39 (95% CI: 0.19–0.98) for CVD onset and 0.23 (95% CI: 0.07–0.70) for stroke compared to the group that engaged in 0–1 type of activity. In contrast, no dose–response relationship between the number of types of leisure-time and household activities and circulatory disease was found among the men.

**Table 4:** Relationship between number of types of leisure-time and household activities and circulatory disease onset

	Men (n = 1343)									Women (n = 2398)								
	0–1 type		2 types			3 or more types				0–1 type		2 types			3 or more types			
	n = 771		n = 314			n = 258			Trend test	n = 927		n = 847			n = 624			Trend test
			HR	95% CI	*P value*	HR	95% CI	*P value*	*P* value			HR	95% CI	*P* value	HR	95% CI	*P* value	*P* value
*CVD*	36			7		11				18		12			8			
Sex and age adjusted model	1.00	(Ref)	0.50	(0.22–1.12)	0.090	0.84	(0.43–1.66)	0.619	0.361	1.00	(Ref)	0.70	(0.34–1.45)	0.334	0.50	(0.22–1.15)	0.101	0.091
Multivariable adjusted model	1.00	(Ref)	0.48	(0.20–1.17)	0.106	0.62	(0.26–1.48)	0.281	0.152	1.00	(Ref)	0.66	(0.30–1.45)	0.300	0.39	(0.19–0.98)	0.046	0.042
*Stroke*	28		4			9				17		10			5			
Sex and age adjusted model	1.00	(Ref)	0.36	(0.13–1.04)	0.059	0.89	(0.42–1.90)	0.765	0.430	1.00	(Ref)	0.62	(0.28–1.35)	0.228	0.33	(0.12–0.89)	0.028	0.022
Multivariable adjusted model	1.00	(Ref)	0.40	(0.13–1.17)	0.094	0.68	(0.26–1.79)	0.431	0.243	1.00	(Ref)	0.54	(0.24–1.25)	0.149	0.23	(0.07–0.70)	0.010	0.006

	Total (n = 3741)								
	0–1 type		2 types			3 or more types			
	n = 1,698		n = 1,161			n = 882			Trend test
			HR	95% CI	*P value*	HR	95% CI	*P value*	*P* value
*CVD*	54		19			19			
Sex and age adjusted model	1.00	(Ref)	0.61	(0.36–1.04)	0.068	0.66	(0.39–1.12)	0.121	0.071
Multivariable adjusted model	1.00	(Ref)	0.51	(0.28–0.94)	0.031	0.49	(0.26–0.93)	0.030	0.013
*Stroke*	45		14			14			
Sex and age adjusted model	1.00	(Ref)	0.52	(0.29–0.96)	0.037	0.57	(0.31–1.04)	0.066	0.032
Multivariable adjusted model	1.00	(Ref)	0.48	(0.24–0.96)	0.039	0.43	(0.20–0.92)	0.030	0..014

Sex and age adjusted model: Adjusted for sex and age

Multivariable adjusted model: sex and age adjusted model + adjusted for smoking (currently, past, never), drinking (currently, past, never, job (y/n), BMI, hypertension (y/n), dyslipidemia(y/n), diabetes (y/n), and physical activity other than during leisure time (sleep, job, transportation, other)

*CVD* cardiovascular disease; *BMI* body mass index; *CI* confidence interval; *HR* hazard ratio

Compared to the group that engaged in 0–1 type of leisure-time and household physical activity after multivariable adjustment, the group that engaged in 2 activity types had a hazard ratio of 0.51 (95% CI: 0.28–0.94) for CVD onset and a value of 0.48 (95% CI: 0.24–0.96) for stroke. Those who engaged in ≥ 3 types of leisure-time and household physical activities had a hazard ratio of 0.49 (95% CI: 0.26–0.93) for CVD onset and 0.43 (95% CI: 0.20–0.90) for stroke.

### Relationship between the number of types of leisure-time and household physical activities and CVD onset

Table 5 presents a forest plot of the results of the Cox proportional hazards models used for the analysis of the relationship between the number of types of leisure-time and household physical activities and CVD onset, by the amount of physical activity. Compared to the group in the lowest tertile, which engaged in 0–1 type of leisure-time and household physical activity even after multivariable adjustment, the participants in the group that engaged in ≥ 2 types of activities and whose level of participation in leisure-time and household physical activity was in the highest tertile had a hazard ratio of 0.31 (95% CI: 0.12–0.79) for CVD onset. Examination of the hazard ratios for stroke indicated that, compared to those who engaged in 0–1 type of leisure-time and household physical activity and were in the lowest tertile, participants who engaged in ≥ 2 types of leisure-time and household physical activities and whose level of participation was in the moderate range had a hazard ratio of 0.38 (95% CI: 0.15–0.95) and those in the highest tertile had a hazard ratio of 0.24 (95% CI: 0.08–0.70).

**Table 5:** Relationship between leisure-time and house hold activities and cardiovascular disease and stroke onset, by number of types, and participation level

			Total (n = 3741)				
			No. of patients	HR	95%CI	*P* value	Interaction test
Types of leisure-time activity							*P* value
*CVD*							
0–1 type	Amount of leisure-time activity_Low	(n = 877)	39	1.00	(Ref)		0.01
	Amount of leisure-time activity_Middle	(n = 519)	10	0.66	(0.27–1.59)	0.354	
	Amount of leisure-time activity_High	(n = 302)	5	0.36	(0.09–1.38)	0.135	
2 or more types	Amount of leisure-time activity_Low	(n = 372)	8	0.45	(0.20–1.04)	0.060	
	Amount of leisure-time activity_Middle	(n = 726)	15	0.48	(0.22–1.04)	0.063	
	Amount of leisure-time activity_High	(n = 945)	15	0.31	(0.12–0.79)	0.015	
*Stroke*							
0–1 type	Amount of leisure time activity_Low	(n = 877)	31	1.00	(Ref)		0.01
	Amount of leisure time activity_Middle	(n = 519)	9	0.62	(0.24–1.63)	0.334	
	Amount of leisure time activity_High	(n = 302)	5	0.33	(0.08–1.36)	0.125	
2 or more type	Amount of leisure time activity_Low	(n = 372)	5	0.39	(0.15–1.02)	0.054	
	Amount of leisure time activity_Middle	(n = 726)	11	0.38	(0.15–0.95)	0.038	
	Amount of leisure time activity_High	(n = 945)	12	0.24	(0.08–0.70)	0.009	

Multivariate-adjusted model—Adjustment factors: sex, age, BMI, hypertension (y/n), diabetes (y/n), dyslipidemia (y/n),

ly drinks/past history of drinking/non-drinker), job (y/n), and physical activities other than during leisure time (sleep, job, transportation, other)

*BMI* body mass index; *HR* hazard ratio; *CI* confidence interval; *CVD* cardiovascular disease

Table 6 shows the results of the cox proportional hazards model of the relationship between the number of types of leisure-time and household activities and the development of CVD, stratified by leisure and household activity time. As a result of stratified analysis, the multivariable adjusted hazard ratio for the development of CVD was 0.55 (95% CI: 0.30–0.98) in Model 1 for the group of people with short leisure time and household and more than two types of leisure and household activities rather than 0–1. However, this was no longer significant in Model 2, which adjusted for BMI, hypertension, dyslipidemia, and diabetes.

**Table 6:** Relationship between the number of types of leisure and household activities stratified by leisure time and household activity and the incidence of cardiovascular diseases

	Low (n = 1872)									
	0–1 types		2 or more types			0–1 types				
	n = 1143		n = 729			n = 555				
			HR	95%CI	*P* value			HR	95%CI	*P* value
*CVD*	46			20		8			18	
Crude	1.00	(Ref)	0.65	(0.39–1.10)	0.112	1.00	(Ref)	0.89	(0.39–2.04)	0.775
Model 1	1.00	(Ref)	0.55	(0.30–0.98)	0.048	1.00	(Ref)	0.78	(0.27–2.22)	0.635
Model 2	1.00	(Ref)	0.56	(0.31–1.01)	0.055	1.00	(Ref)	0.69	(0.23–2.05)	0.507

Model 1: Adjusted for sex, age, smoking (currently, past, never), drinking (currently, past, never), and job (y/n)

Model 2: Model 1 + adjusted for BMI, hypertension (y/n), dyslipidemia (y/n), and diabetes (y/n)

## Discussion

In this longitudinal study, conducted among individuals in the Japanese general population, the level of participation in leisure-time and household physical activity was inversely related to new-onset CVD among women. In addition, a significant lowering of the CVD risk was recognized in association with the increase in the number of types of leisure and household activity. We found no relationship between new-onset CVD and either the level of participation or the number of leisure-time and household physical activity types among men.

In this study, we investigated new-onset CVD in association with not only the amount of leisure-time and household physical activity engaged in but also the number of leisure-time and household physical activity types that were participated in. A previous meta-analysis showed that the amount of physical activity participated in was associated with stroke risk [26]. Another meta-analysis analyzed 21 articles with more than 1,000 subjects and more than 5 years of follow-up among prospective cohort studies examining the relationship between physical activity and CVD incidence from 1980 to 2010, and found that the risk of death from CVD was significantly lower in the group with the highest leisure-time physical activity compared to the group with the lowest leisure-time physical activity. The risk of death from CVD was reported to be significantly lower for both men and women in the highest leisure time group compared to the lowest leisure time group (hazard ratio: 0.76 and 0.73, respectively) [27]. The results of the aforementioned meta-analyses are consistent, in that they showed the presence of an inverse relationship between the amount of physical activity during leisure time and the risk of CVD mortality. Previous studies conducted in Japan have suggested that the risk of CVD mortality reduces with increases in the number of days/week of leisure-time physical activity participation [28]. The results of the present study are generally consistent with those of previous studies.

The mechanism through which participation in physical activity prevents CVD includes improvements in the level of receptiveness to insulin and lipoprotein metabolism. The results of the National Health and Nutrition Survey conducted in Japan indicated that increases in the number of steps walked and HDL-C levels were positively correlated, whereas an inverse correlation was noted in terms of TG levels [29]. A meta-analysis that compared the effect of aerobic exercise on middle-aged and elderly individuals compared to that in a group that did not participate in exercise found that engagement in exercise decreased the levels of low-density lipoprotein cholesterol and TG as well as increased the levels of HDL-C [29]. Exercise activates lipoprotein lipase, promotes the breakdown and use of TGs, and inhibits the degree of decrease in vascular endothelial function. In addition, participation in regular leisure-time physical activities, such as exercise, improves vascular endothelial function [30, 31], and regular engagement in aerobic exercises, such as jogging and walking, leads to decreases in the systolic BP (by 3.84 mmHg) and diastolic BP (by 2.58 mmHg) [32]. The mechanism involved in the prevention of cerebrovascular disease includes improvements in the quality of cardiovascular function, and secondary actions that work via improvements in the quality of cerebrovascular disease risk factors, such as hypertension, diabetes, lipedema, obesity, and atrial fibrillation [33].

A Cox proportional hazards model was performed on the association between the number of types of leisure-time and household activities and the risk of developing CVD by stratifying leisure and household activity time. The results showed that a combination of two or more types of activities might have a higher preventive effect than 0–1 type in the group with short activity time. However, after adjusting for morbid factors such as obesity, hypertension, dyslipidemia, and diabetes, the results became insignificant. These morbid factors have been reported to be associated with CVD in previous studies [34–36]. Therefore, we hypothesized that the number of types of leisure and household physical activities would influence the development of CVD through these pathological factors. A previous study [37] reported that, whereas resistance training and aerobic exercise both act to improve the quality of vascular endothelial function, participation in a combination of both results in a synergistic effect. Another study found that the combination of resistance training and aerobic exercise lowered type 2 diabetes patients’ HbA1c levels by 0.62% and the fasting blood sugar level by 35.8 mg/dL, compared to resistance training alone; however, compared to aerobic exercise alone, the HbA1c level decreased by 0.17% and the fasting blood sugar level decreased by 10.6 mg/dL [38]. Since this study was not able to consider the type or combination of leisure activities, it is unclear whether the participants were engaged in aerobic exercise or resistance training. Therefore, it is not possible to elucidate the physiology from the results of this study, but we speculated that people who engage in two or more types of leisure and household activities may reduce not only mental stress but also physical stress and the load on blood vessels, which may help prevent the onset of cardiovascular disease.

We did not observe a relationship between the amount and number of types of leisure-time and household physical activities and new-onset CVD in men. A European cohort study [39], likewise, found that participation in moderate-intensity leisure-time physical activity decreased a woman’s risk of cerebrovascular disease; however, in men, leisure-time physical activity and cerebrovascular disease were unrelated. Some previous studies identified a sex-based difference in the relationship between leisure-time physical activity and cerebrovascular disease. Schnall et al. [40] reported that, among men with high levels of work-related stress, the BP levels during work were high, rendering them liable to the experience of progressive hypertrophy of the heart. The results of another study [41] suggested that, in contrast to the beneficial health effects obtained in association with leisure-time physical activity, work-related physical activity may be harmful to health, which is the opposite effect of two or more types of leisure time physical activity on the health. Men tend to spend longer hours at their workplace and have only a superficial relationship with their community; thus, once they retire, the specific types of physical activity they engage in (balance between work-related physical activity and leisure-time physical activity) change drastically. In the present study, 57.2% of the male participants were working and many of them engaged in large amounts of physical activity at work. Thus, we believe that the benefits, in terms of the protection against CVD offered by participation in leisure-time and household physical activity, may have been cancelled by the presence of work-related stress.

The strength of this study is that it was possible to evaluate the amount of physical activity by classifying it into domains that cannot be evaluated by the physical activity meter. It also takes into account sitting behavior, which is known as an independent risk of cardiovascular disease. In addition, it follows up on previous results on leisure-time and household physical activity and risk of developing CVD, but also includes a new number of types of leisure-time and household physical activities. However, the present study has some limitations. First, since the study sample comprised individuals who underwent regular health checkups and special health checkups, the presence of selection bias cannot be ruled out. Second, as only a small number of AMI events were present, our investigation of the relationship between leisure-time and household physical activity and AMI onset was insufficient. Third, it is a well known fact that smoking and alcohol consumption are strongly correlated with the development of CVD. In the present study, smoking and alcohol consumption could be classified into only three categories each, and therefore, caution should be exercised in their interpretation. Fourth, individuals who undertake a lot of leisure and household activities may have other health consciousness. Fifth, this study does not consider the intensity of physical activity. In the future, detailed analysis considering physical activity time and intensity of each activity is required. Sixth, this study combined housework activities and leisure activities, and it was not possible to clarify which had the stronger influence. Therefore, caution must be exercised in the generalization of the results of this study.

In conclusion, the results of this longitudinal study of the Japanese general population indicated the presence of an inverse relationship between the level of participation in leisure-time and household physical activity and new-onset CVD. Our investigation of the types of leisure-time and household physical activities indicated the possibility that engagement in two or more types of activities may protect against CVD onset, compared to participation in 0–1 type of activity. In recent years, there have been increasing reports that the accumulation of light-intensity physical activity brings health benefits [42–44], indicating the importance of accumulating leisure activities and household physical activity. In the future, further detailed analyses of the combinations of leisure-time and household physical activities are needed for the formulation of strategies aimed at CVD prevention.

## Data Availability

The datasets analyzed during the current study are not publicly available due to them containing information that could compromise research participant privacy and consent, and ethical restrictions. The data are available from YK upon reasonable request within　the limitations of informed consent by the research committee of the Takashima Study upon acceptance. Every request will be reviewed by the Institutional Review Boards of Shiga University of Medical Science and Tsuruga Nursing University. After approval, the researchers can access the data according to the approval conditions.

## Abbreviations

BMI: Body mass index
BP: Blood pressure
TG: Triglyceride
HDL-C: High-density lipoprotein cholesterol
CVD: Cardiovascular disease
AMI: Acute myocardial infarction
CI: Confidence interval
MET: Metabolic equivalent
HbA1c: Glycated hemoglobin
JALSPAQ: Japan Arteriosclerosis Longitudinal Study Physical Activity Questionnaire
ICD: International Classification of Diseases
HHS: Health and Human Services
